# Whose Knowledge Heals? Transforming Teaching in the Struggle for Health Equity

**DOI:** 10.1177/10901981231177095

**Published:** 2023-08-01

**Authors:** Erin Manalo-Pedro, Katrina M. Walsemann, Gilbert C. Gee

**Affiliations:** 1 University of California, Los Angeles, Los Angeles, CA, USA; 2 University of Maryland, College Park, MD, USA

**Keywords:** graduate training, racism, health disparities, justice, pedagogy, curriculum

## Abstract

Racial health inequities persist despite many attempts to correct them. Inadequate comprehension of racism obscures the ordinariness of racism in public health institutions. In addition to applying critical race theory (CRT) to the research and practice of public health, we argue that the struggle for health equity must also apply CRT toward the *teaching* of public health students. Adhering to conventional approaches in academic public health without grappling with their roots in Whiteness reproduces a public health workforce that is insufficiently equipped to address the complex, systemic issues underlying health inequities. By default, academic public health excludes the perspectives of scholars of color, relies too heavily on theories of individual behavior, and applies top-down teaching methods. To make durable changes, the rising generation of public health scholars and practitioners must understand how health equity fits within broader struggles for racial and social justice. Thus, we critique three responsibilities for teaching about public health: assigning readings, shaping analytical lenses with theories, and modeling change through andragogy. By questioning whose knowledge is legitimized when defining public health needs, whose lenses are used to prioritize solutions, and whose insights drive change, we can train a public health workforce more critical of racism, and more prepared to deal with the enduring reality of racial relations.

“Reality is not fixed, not a given. Rather, we construct it through conversations, through our lives together.”—Richard Delgado, 1989

In 2021, public health organizations throughout the United States declared racism “a serious public health threat” in response to the Movement for Black Lives (Walensky, 2021). However, Black, Indigenous, and People of Color (BIPOC) scholars have articulated this stance for decades (Williams & Collins, 1995). What stalls racism-conscious practice?

One postulation is the lack of training on racism (Shaw-Ridley & Ridley, 2010). Spurred by student-led efforts at the University of Washington, discussing racism as a challenge to health equity was formalized as a Master of Public Health (MPH) competency for accreditation by the Council on Education for Public Health in 2016 (Hagopian et al., 2018). Still, schools and programs of public health inadequately prepare graduates for anti-racist public health research and practice (Cross, 2018; Komro et al., 2018).

Critical race theory (CRT) prompts us to ask why racial health inequities persist. While Public Health Critical Race Praxis (PHCRP) guides the *research and practice* of public health (Ford et al., 2019), we argue that examining the *teaching* of public health students can broaden our understanding of the struggle for health equity. Guided by CRT of education (Delgado, 1989; Delgado Bernal & Villalpando, 2002; Yosso, 2005), we, as racial health equity scholars, question whose knowledge is taught in public health.

## Racism, Whiteness, and CRT of Education

Racism systematically benefits White supremacy at the expense of BIPOC (C. P. Jones, 2018). This racial hierarchy is upheld, in part, by curtailed disciplinary self-critique (Bowleg, 2021; Liburd et al., 2019). We invoke the five tenets from CRT of education (Solórzano & Yosso, 2001) to elucidate academic public health’s entanglements with racism.

*1. The centrality of race and racism and their intersectionality with other forms of subordination.* CRT addresses racism as the primary form of oppression which, interacting with patriarchy and capitalism, stratifies access to education, wellness, and economic security. The challenge of training racism-conscious public health graduate students becomes clearer upon acknowledging long-held racist views in public health history. For example, public health concerns regarding the “hybrid of the most despicable, a mongrel of the most detestable that has ever afflicted the earth” (Volpp, 2000, pp. 801–802) justified bans on interracial marriage. Similar depictions of non-White people as biologically inferior substantiated eugenic policies, such as exclusionary immigration and forced sterilizations (Chowkwanyun, 2011; Stern, 2005). Thus, dismantling racism demands confronting contemporary public health fundamentals developed through racist legacies.
*2. The challenge to dominant ideology.* The racial hierarchy which positions White people above others is perceived as natural (Bonilla-Silva, 1997, 2012). Legal scholar Cheryl Harris (1993) described the essence of Whiteness as “the legal legitimation of expectations of power and control that enshrine the status quo as a neutral baseline, while masking the maintenance of white privilege and domination” (p. 1715). This baseline is achieved through “centering,” the dynamic where certain perspectives, most often those of White cismen (whose gender identity aligns with assigned sex at birth), are upheld as the “norm” by which others are compared (J. M. Jones, 1988).
 CRT renders the hidden structures that maintain racial hierarchy more visible to facilitate their undoing. Defaulting to Whites as the “reference group” in disparities research implies White is normative (C. P. Jones et al., 1991) which obscures the health advantages conferred by White privilege (Sullivan, 2014). Consider the situation where a professor describes the readings on the Framingham cohort as “classics,” and then talks about Tongans as “culturally different” and “underprivileged.” Alternatively, they could have referred to White people and culture as “privileged” (Yosso, 2005). However, doing so feels odd because it disrupts the prevailing assumption that minoritized communities are deviant.
*3. The commitment to social justice.* CRT emphasizes dismantling racism and other root causes of inequities. By generating a shared understanding of reality, CRT facilitates the conditions to transform society (Delgado, 1989). Critical educators develop students’ critiques of oppression and motivation for social justice to collaboratively engage in transformational resistance (Solórzano & Bernal, 2001).
*4. The centrality of experiential knowledge.* CRT values lived experience. The margins refer to a social location wherein BIPOC communities generate unique perspectives on oppression and resistance (Hooks, 1990; Sweet, 2020). However, public health enthusiasm for collecting *data* from BIPOC communities overshadows support for investing in *students* from BIPOC communities as prospective researchers (Pasick et al., 2012). Notwithstanding modest increases in the percentage of BIPOC doctoral graduates, centering inimitable insights from BIPOC students and scholars in the predominantly White field of public health remains a struggle (Goodman et al., 2020; Pasick et al., 2012).
*5. The transdisciplinary perspective.* CRT provides sociopolitical and historical context to health inequities by drawing from multiple disciplines, including law, history, ethnic, and gender studies (Solórzano & Yosso, 2001). While civil rights, multiculturalism, and health disparities approaches superficially reform the status quo in law, education, and public health, respectively, the CRT perspective argues for radical transformation of these systems (Bell, 1980; Ford & Airhihenbuwa, 2018; Ladson-Billings & Tate, 1995; Solórzano & Bernal, 2001).

CRT of education clarifies how teaching conventionally centers Whiteness as the unquestioned standard. Its tenets inform our critique of common teaching responsibilities as everyday opportunities for advancing health equity. To eliminate racial health inequities, public health educators must deliberately transform teaching to value voices from the BIPOC community (Bowleg, 2021).

## Transforming Teaching

We organize our critique around three ordinary responsibilities of public health educators—assigning readings, shaping analytical lenses, and modeling change through andragogy—as opportunities to name racism, question how it operates, and organize to dismantle it (C. P. Jones, 2018). First, educators select readings for syllabi which structures how students define public health needs. Because how one defines a problem guides strategies to respond, students’ aptitudes to identify racism as determinants of health hinge on including BIPOC authors and critical epistemologies that challenge dominant narratives in course reading lists. Second, educators shape how students analyze the causes of health inequities by curating which theories are taught. To interrogate how racism operates, students must critically interpret the mundane encounters between BIPOC and the systems that structure life (Gee et al., 2019). Third, educators model decision-making through andragogy, how they engage adult learners. To develop the commitment to justice and practical skills required to organize and strategize against racism, educators and students must cocreate classroom environments that amplify BIPOC students’ aspirations for change.

We urge educators to decenter Whiteness when not only teaching *about* racial health inequities but also *throughout* public health graduate training. That is, readings, theories, and andragogy represent everyday opportunities to transform *teaching* by *learning* from BIPOC scholars, community members, and students. Furthermore, “because those with the power to change institutions were also educated by these institutions . . . they . . . often perform their lives devoid of racial consciousness” (Patton, 2016, p. 234). Table 1 outlines how readings, theories, and andragogy serve as opportunities to either uphold the status quo (conventional competencies) or challenge it (CRT-informed competencies).

**Table 1.** Comparison of Select Conventional and Critical Race Theory (CRT) Learning Competencies for Academic Public Health.

Educator responsibility	Conventional competency^a^	CRT-informed competency	Example of CRT-informed teaching
*Constructing reality through assigned readings*: Teach the canon assuming it represents objective truth	Identify basic theories, concepts, and models from a range of social and behavioral disciplines that are used in public health research and practice.	Identify theories, concepts, and models that describe social and health inequities from nondominant perspectives.	Incorporate readings by BIPOC authors in syllabi. Discuss whether/how the author’s positionality nuances dominant ideology.
	Describe the roles of history, power, privilege, and structural inequality in producing health disparities.	Describe the historical and contemporary influence of dominant theories, concepts, and models used in public health research and practice in perpetuating health inequities.	Assign case studies on the eugenic origins of contemporary health policies and practices (e.g., sterilization, statistics).
	Explain how professional ethics and practices relate to equity and accountability in diverse community settings.	Discuss the implications of decentering Whiteness for health equity.	Provide examples of research articles that do not use White as the reference group.
*Shaping analytical lenses with theories*: Focus on individual behavior	Identify the causes of social and behavioral factors that affect health of individuals and populations.	Distinguish between color-evasive and racism-conscious explanations for health differences.	Provide opportunities to practice identifying manifestations of cultural and structural racism.
	Identify individual, organizational, and community concerns, assets resources, and deficits for social and behavioral science interventions.	Explain how the ideology of meritocracy and the focus on individual behavior stymie structural interventions.	Assign case studies applying structural root cause analyses to common public health topics.
	Explain how the contexts of gender, race, poverty, history, migration, and culture are important in the design of interventions within public health systems.	Explain how the history of sexism, racism, capitalism, colonialism, and imperialism shape contemporary access to health-enhancing resources at the local, state, national, transnational, and planetary levels.	Teach transdisciplinary theories, concepts, and models that examine sociohistorical, political pathways to health inequities.
	Describe the roles of history, power, privilege, and structural inequality in producing health disparities.	Identify local examples of community-led initiatives to change structures that produce health inequities.	Invite community organizers as guest speakers to discuss successful campaigns.
	Analyze the effects of political, social, and economic policies on public health systems at the local, state, national, and international levels.	Develop public health programs and strategies that disrupt the structures that produce health disparities.	Partner with community organizations to develop opportunities for students to engage in action research.
*Modeling change through andragogy*: Treat students as empty vessels using didactic teaching	Explain why cultural competence alone cannot address health disparity.	Explain how dominant groups maintain power relations through cultural capital.	Examine how Whiteness is handled in the classroom.
	Engage in dialogue and learning from others to advance public health goals. Demonstrate team building, negotiation, and conflict management skills.	Engage in dialogue and learning from others to generate authentic relationships with peers and community members.	Cultivate a sense of community in the classroom through shared vulnerability and cultural humility. Model reflexivity by sharing how the instructor’s positionality shapes assumptions and how to address them.
	Use the basic concepts and skills involved in culturally appropriate community engagement and empowerment with diverse communities. Apply the principles of community-based participatory research to improve health in diverse populations. Appreciate the importance of working collaboratively with diverse communities and constituencies (e.g., researchers, practitioners, agencies, and organizations).	Recognize students’ positionality within multiple power relations through reflexivity. Apply personal and professional knowledge to generate innovative solutions for long-standing inequities. Identify and appreciate students’, peers’, and community members’ community cultural wealth.	Legitimate lived experience and community cultural wealth as valid ways of knowing. Incorporate assignments that ask students to reflect on what they or their families already know or have experienced for a particular health issue or topic. Partner with community organizations to develop opportunities for students to engage in action research. Incorporate the option for arts-based projects to promote creativity and acknowledge other forms of expression.
	Discuss the importance and characteristics of a sustainable diverse public health workforce.	Identify and work to disrupt systemic policies and practices that push BIPOC students out of the public health workforce.	Routinely provide students with microaffirmations, which are subtle, everyday acts that communicate that students belong. Refer students to appropriate campus resources for further identity-based wellness and/or basic needs support.

*Note*. BIPOC = Black, Indigenous, and People of Color.

a Examples of conventional learning competencies were obtained from https://www.aspph.org/teach-research/models/mph-competency-model/.

### Constructing Reality Through Assigned Readings

Class readings reinforce what is considered valued and normative in a field. In public health, reading lists transmit ways to think about public health needs (Schucan Bird & Pitman, 2020). When instructors assign readings, they exercise the power to set students’ foundational knowledge of public health. As intellectual currency, citations simultaneously facilitate the dissemination of some knowledge while restricting that of others (Bowleg, 2021).

Public health reading lists often cite established scholars who, given historical and ongoing racism and sexism in the academy, are most often White men (Ford & Airhihenbuwa, 2018; Zidani, 2021). The presumed superiority of Western knowledge has propagated through centuries of colonization, rupturing, denigrating, and erasing Indigenous ways of knowing (Smith, 2012). The “apartheid of knowledge” in academia “legitimates” mainstream knowledge while discrediting BIPOC epistemologies (Delgado Bernal & Villalpando, 2002). This cyclical devaluation of insights impedes progress in the pipeline of BIPOC educators, researchers, and authors (Bowleg, 2021; Yancey et al., 2007). Unequal opportunities to publish critical research and design related courses yield structural barriers to spreading BIPOC knowledge.

Thus, a predominantly White faculty defaults to crafting syllabi which center publications authored by White scholars as the public health “canon.” For example, authors of assigned articles from two academic years of syllabi in a London MPH program overwhelmingly skewed toward institutions in the United States and United Kingdom (Price et al., 2022). Similarly, content analysis of 30 syllabi from top social/behavioral science MPH programs in the United States showed that all 15 of the most commonly taught theories were first-authored by White men and the two most commonly assigned behavioral theory textbooks were coedited by White women (Harvey & McGladrey, 2019).^1^ Centering dominant perspectives naturalizes racist conditions and constrains students’ ingenuity (Kagawa-Singer, 2000).

What is excluded from syllabi also maintains health inequities (Petteway, 2020). To prepare students to critically identify public health issues, educators should prompt students to consider *whose* definitions of health are centered (Chandanabhumma et al., 2020; Delgado, 1989). Rather than assuming one universal truth, CRT appreciates the social construction of knowledge production and, relatedly, values BIPOC epistemologies (Ford & Airhihenbuwa, 2010). As Hill Collins (1990, p. 269) wrote, “[T]he significance of a Black feminist epistemology may lie in its ability to enrich our understanding of how subordinate groups create knowledge that fosters both their empowerment and social justice.” Because people at the margins of society uniquely understand oppression and resistance, educators should incorporate texts authored by BIPOC scholars that can inform pathways to health equity (Bowleg, 2021; Patton, 2016).

Racism-conscious educators can redistribute power by constructing syllabi for racial health equity rather than default to previously used materials. Table 1 provides examples of CRT-informed competencies and relevant teaching practices that promote disciplinary self-critique. Isolated modules on racism or infrequently offered electives are necessary but insufficient. Instead, BIPOC scholarship must be incorporated into curricula on numerous foundational public health topics, as illustrated in Table 2. In core courses, educators can illustrate key questions in the field without defaulting to the “canon” (Zidani, 2021) and encourage students to examine authors’ potential biases (Bowleg, 2017). Educators are responsible for transmitting ideas through syllabi; whose knowledge is legitimized?

**Table 2.** Examples of BIPOC Scholarship on Foundational Public Health Topics.

Public health topic	Relevant articles with BIPOC first authors
Research methodology	Agénor, 2020; Bowleg, 2017; Ford et al., 2018; LaVeist, 1994; Petteway et al., 2019
Health policy	Barlow & Johnson, 2021; Hardeman et al., 2022
Program development and implementation	Chandanabhumma & Narasimhan, 2020; Shelton et al., 2021; Walters et al., 2020
Demography	Soon et al., 2020; Zuberi, 2000
Built environment	García et al., 2016; Wang et al., 2019
Climate change	Ratima et al., 2019
Reproductive justice	Loder et al., 2020
Nutrition	Hite & Carter, 2019
Physical health	Walters et al., 2011
Behavioral health	Misra et al., 2022; Wahbi & Beletsky, 2022

*Note*. These examples are not exhaustive. BIPOC = Black, Indigenous, and People of Color.

### Shaping Analytical Lenses With Theories

Theory informs public health research and practice (Krieger, 2014). Although earlier health promotion initiatives focused on individual behavior change (Becker, 1986), health equity scholars recommend structural analyses to ascertain how upstream systems of power obscure multiple, complex pathways to health justice (Braveman & Parker Dominguez, 2021; Krieger, 2020). Choosing which theories to teach either stifles or enhances students’ abilities to connect structural racism to health inequities.

Individual behavior theories perpetuate Whiteness by utilizing dominant ideologies of meritocracy and color-evasiveness to explain population health disparities (Bonini & Matias, 2021; Kagawa-Singer, 2000; Neely et al., 2020). Among the 10 most commonly taught social/behavior theories in MPH programs, eight focus on individual factors (Harvey & McGladrey, 2019). Yet individual-level approaches are not well suited to address structural problems (Golden & Earp, 2012).

Controlling for individuals’ “race” stymies progress for measuring exposures to racism (Ford et al., 2019; LaVeist, 1994). Health equity experts theorize a throughline between power structures, differential access to resources, unequal exposure to harmful conditions, and physiological responses (Braveman & Parker Dominguez, 2021; Tsai et al., 2021). Yet explicit theory, structural or otherwise, is rarely invoked to explain empirical racial health inequities research in high-impact journals, limiting students’ access to nuanced analyses (Krieger et al., 2021; Mannor & Malcoe, 2022). To disrupt myths about individual responsibility for health, educators should incorporate transdisciplinary theories to reveal distal determinants, sharpen students’ analysis of Whiteness, and deepen appreciation for BIPOC epistemologies (Neely et al., 2020; Sabado-Liwag et al., 2022; Sun, 2014). Table 1 Row 2 suggests ways to incorporate such analytical lenses into teaching.

To improve conceptualizations of structural oppression, educators must teach students various analytical approaches using CRT lenses (Ford et al., 2019; Hagopian et al., 2018 C. P. (Jones, 2018; Neely et al., 2020). PHCRP provides an ordered process to guide racism-conscious empirical research and action-oriented practice for racial health equity (Ford & Airhihenbuwa, 2010). PHCRP scholars use tools from multiple disciplines to trace the roots of contemporary health outcomes, as noted in Table 3. Theories about structural determinants of inequity have been recommended elsewhere (Harvey, 2020).

**Table 3.** Transdisciplinary Empirical Analyses of Public Health Issues.

Analytical tools	Public health issue
Autoethnography	Historical trauma, community resilience, and healing (Cacari Stone et al., 2021)
Critical discourse analysis	Nutrition guidelines (Hite & Carter, 2019)
Deconstruction of norms	Social support (Huynh, 2022)
Historical case study	Health services (Yang, 2010)
Land-based healing	Health promotion (Johnson-Jennings et al., 2020)
Oral histories	Community health assessment (Hernandez et al., 2017)
Policy surveillance	Racial equity environment (Agénor et al., 2021)
Regression with intersectional lens	Worker health (Nazareno et al., 2021)

*Note*. These examples are not exhaustive.

Students can hone their cultural intuition to expose White supremacy in the ordinary lives of BIPOC (Delgado Bernal, 1998; Ford & Airhihenbuwa, 2010). Given the variants of racism (Davis, 2021), scholars of CRT in education have more precisely articulated its tenets for specific groups: LatCrit (Solórzano & Yosso, 2001), TribalCrit (Brayboy, 2005), AsianCrit (Iftikar & Museus, 2018), DisCrit (Annamma et al., 2017), and UndocuCrit (Aguilar, 2019). These branches distinguish racialized exposures to harm.

Public health students must also recognize how racism intersects with other systems of oppression (Agénor, 2020), including capitalism (Laster Pirtle, 2020), patriarchy (Bowleg, 2017), colonialism (Chandanabhumma & Narasimhan, 2020), and ableism (Annamma et al., 2017). For example, conventional public health may attribute elevated rates of obesity among low-income communities of color to limited access to well-equipped parks. While plausible, racial capitalism further unveils more than a century of urban planning decisions to privatize recreational spaces (e.g., single-family homes with backyards), destroy non-White communities (e.g., developing freeways through Black, Latinx, or Asian neighborhoods), and maintain disinvestment in public parks (e.g., property tax policies) (García et al., 2016). Supplanting home-based physical fitness education, the enhanced inquiry proposes anti-capitalist community organizing to sustainably invest in healthier neighborhoods for communities of color.

Analytical transformation requires shifting perceptions of community members from “at-risk” study participants to collaborators with self-determined priorities (Smith, 2012; Tuck, 2009). As the experts of their own lives (Chandanabhumma et al., 2020), BIPOC community members should be involved in setting the agenda in academic public health, including informing curricular decisions (Hardeman et al., 2018; Perez et al., 2021). Public health programs are responsible for training students to recognize the determinants of health; whose lenses are used?

### Modeling Change Through Andragogy

Educators’ interactions with students affect the latter’s capacity for change as future public health professionals (Chávez et al., 2006). Public health educators must not only facilitate knowledge acquisition but also, as an applied field, prepare graduates to cocreate the conditions for health equity (Komro et al., 2018). Whether “pedagogy” for children or “andragogy” for adults, teaching strategies affect students’ potential to change society (Freire, 2000; Solórzano, 2022). To encourage students’ agency, educators must make andragogical decisions that affirm, not dehumanize, as described in Table 1 Row 3.

Anti-racist andragogy refers to “instructional approaches in adult education designed to interrupt systems of oppression and challenge racist and discriminatory ideologies” (Sandifer et al., 2022, p. 1). Traditionally, Malcolm Knowles’ concept of andragogy acknowledges that adults bring a wealth of experience to the classroom, which shapes their comprehension of material. From an anti-racist perspective, this suggests that instructors should actively encourage students to reflect on their experiences as integral to dismantling racism.

Public health graduate educators, however, frequently utilize top-down, didactic methods that view students as empty vessels for depositing knowledge, a style critical education philosopher Paulo Freire referred to as “banking” (Chávez et al., 2006; Freire, 2000). This structure of teaching is inherently hierarchical, with professors serving as gatekeepers of knowledge and opportunities. For example, the presumed default is to send course syllabi as PDFs as opposed to collaborative, modifiable documents. The unidirectionality of power relations is normalized throughout the course (e.g., enrollment requisites, classroom engagement, standardized assignments, office hours).

Power dynamics typically maintain a culture of Whiteness that dehumanizes BIPOC students by divorcing content from lived experiences (Camangian & Stovall, 2022; Ladson-Billings, 1995). Engaged dialogue on epistemology enables students to interpret the world in context of their social identities and professional commitments. When educators fail to connect subject matter to students’ knowledge from their lives, BIPOC students’ assets frequently remain unrecognized (Delima, 2019; Yosso, 2005).

When White culture is synonymous with the norms in academic public health, students familiar with the dominant culture benefit (Utt, 2018). For example, “dispassionate” neutrality is often conflated with professionalism, socializing graduate students of color to silence their emotions to uphold the semblance of objectivity (Bowleg, 2021; Petteway, 2021; Solórzano & Yosso, 2001). Relatedly, requiring adherence to “academically acceptable English” prioritizes language that is “palatable to mainstream white society,” often diverging from BIPOC students’ preferred communication styles (Camangian & Stovall, 2022). In addition, environments lacking familiar sights, sounds, scents, and tastes contribute to feelings of exclusion (Chávez et al., 2006; Yancey et al., 2007). By teaching how they were taught, educators may unintentionally inflict psychological harm and distract students who respond by organizing others (Gwayi-Chore et al., 2021; McSorley et al., 2021).

In contrast, Freirean educators informed by CRT collaborate with students by incorporating humanizing activities toward social change (Camangian & Cariaga, 2021; Chávez et al., 2006). As a precursor to regarding students as cocreators of knowledge, educators must be mindful of their own relationships to power (Petteway, 2021; Rodney, 2016), specifically their complicity in upholding Whiteness (Aqil et al., 2021; Bonini & Matias, 2021). Reflecting on teaching philosophies and privileges can reveal underlying biases about what scholars look like, where they have been trained, and whose realities are deemed “unworthy of intellectual interrogation” (Camangian & Stovall, 2022; Posselt, 2018). Rejecting the caricature of the all-knowing professor, Freirean educators embrace collective dialogue as an ongoing process of learning alongside students with vulnerability, humility, and courage (Solórzano, 2022).

To engage in the reflexivity required to contribute a shared understanding of health inequities (McSorley et al., 2021), students should be liberated from rote demonstrations of knowledge. Narrative storytelling may resonate more deeply among students with strong oral traditions; indeed, testimonios have been framed as a critical race strategy for addressing segregated knowledge (Huber, 2009). Reflection journals and arts-based projects creatively integrate students’ professional and political voices (Griffith & Semlow, 2020; Lightfoot et al., 2021; Petteway, 2021). The insights generated through these activities could inform collaborative training opportunities for action research financially supported by local public health organizations (Blenner et al., 2021). Health equity cannot be achieved in silos (Galea & Vaughan, 2019). Educators are responsible for training students to ensure the public’s health; whose insights are driving change?

CRT challenges claims of neutrality. Educators should abandon conventional approaches to teaching public health to unleash our collective potential to dismantle White supremacy. Transformation may begin with discomfort (Chávez et al., 2006), particularly among those who benefit from Whiteness (Leonardo & Porter, 2010). Unlearning oppressive ideologies may feel strange and unsettling, given the myriad of societal norms which elevate educators above students, naturalize racial hierarchies, and depoliticize death. Ultimately, the goal for transforming teaching is humanization, changing relations by changing standards of who is seen as fully human (Leonardo & Porter, 2010) and embracing discomfort and struggle as necessary for growth (Rodney, 2016). Racism-conscious teaching commits to broadening perspectives, advancing structural analyses, and sustaining transformative resistance.

## Public Health Implications

Public health training must evolve to match transformations in public health practice. For example, equity-focused measures have been incorporated into the 10 essential public health services for health department accreditation (The Futures Initiative, 2020). For community health improvement process development, departments must demonstrate engagement with multiple community organizations and review local impacts on exclusionary policies (e.g., redlining) and disinvestment (Public Health Accreditation Board, 2022). To train the critical, anti-racist professionals needed to equitably assure these services, we need critical, anti-racist health workforce development (Bonini & Matias, 2021; French et al., 2020; Maglalang & Rao, 2021; Ramirez-Valles et al., 2022; Tsai et al., 2021).

Relatedly, the socialization of health professionals should be incorporated into research on structural determinants of health inequity. That is, health equity scholars could monitor trainees’ preparedness for equity. For example, course syllabi could be analyzed to detect differences in assigned readings (e.g., author race, research paradigms, and theories). What is taught could be compared across institutional types (e.g., minority-serving institutions), assessed longitudinally (Price et al., 2022), or juxtaposed against local demographic composition (Lucy et al., 2020). Schools and programs in public health have begun to transparently discuss struggles and innovative approaches for centering equity in curricular policies (Hagopian et al., 2018; Perez et al., 2021; Seiler et al., 2022). Training program evaluations should operationalize concepts from CRT of education (e.g., microaffirmations, community cultural wealth, transformational resistance) to garner support for humanizing andragogy (Neely et al., 2020; Pasick et al., 2012; Solórzano, 2022).

Public health educators must acknowledge the futility of using White logic and White methods to change health inequities produced by Whiteness (Bowleg, 2021; Patton, 2016; Stewart, 2003). Instead, we need a public health workforce that can define needs informed by BIPOC scholarship, substantiate analyses through the material realities of BIPOC lives, and collectively build new futures inspired by BIPOC aspirations. A CRT approach to teaching would provide students with the knowledge, analytical skills, and collaborative networks necessary for organizing anti-racist partnerships for racial health equity (Mitchell et al., 2022; Neely et al., 2020).

Transforming teaching for health equity is not a prescription for specific readings, theories, or course assignments. Rather, by critically questioning whose knowledge is legitimized when determining public health needs, whose lenses are used to prioritize solutions, and whose insights drive change, we can train a public health workforce more critical of racism, and more prepared to deal with the complexities and contradictions of racial relations. To manage the serious public health threat of racism, we must commit to asking ourselves, whose knowledge heals?

## Notes

1. Harvey and McGladrey published the list of theories with their authors. We estimated the racial and gender composition of these authors through publicly available biographical data and photographs.

## References

[bibr1-10901981231177095] Agénor M. (2020). Future directions for incorporating intersectionality into quantitative population health research. American Journal of Public Health, 110(6), 803–806. 10.2105/AJPH.2020.305610 32298180 PMC7204457

[bibr2-10901981231177095] Agénor M. Perkins C. Stamoulis C. Hall R. D. Samnaliev M. Berland S. Bryn Austin S. (2021). Developing a database of structural racism–related state laws for health equity research and practice in the United States. Public Health Reports, 136(4), 428–440. 10.1177/0033354920984168 33617383 PMC8203034

[bibr3-10901981231177095] Aguilar C. (2019). Undocumented critical theory. Cultural Studies—Critical Methodologies, 19(3), 152–160. 10.1177/1532708618817911

[bibr4-10901981231177095] Annamma S. A. Jackson D. D. Morrison D. (2017). Conceptualizing color-evasiveness: Using dis/ability critical race theory to expand a color-blind racial ideology in education and society. Race Ethnicity and Education, 20(2), 147–162. 10.1080/13613324.2016.1248837

[bibr5-10901981231177095] Aqil A. R. Malik M. Jacques K. A. Lee K. Parker L. J. Kennedy C. E. Mooney G. German D. (2021). Engaging in anti-oppressive public health teaching: Challenges and recommendations. Pedagogy in Health Promotion, 7(4), 344–353. 10.1177/23733799211045407

[bibr6-10901981231177095] Barlow J. N. Johnson B. M. (2021). Listen to Black women: Do Black feminist and womanist health policy analyses. Women’s Health Issues, 31(2), 91–95. 10.1016/j.whi.2020.11.001 33229295

[bibr7-10901981231177095] Becker M. H. (1986). The tyranny of health promotion. Public Health Reviews, 14(1), 15–23. 3775038

[bibr8-10901981231177095] Bell D. A. (1980). Brown v. Board of education and the interest-convergence dilemma. Harvard Law Review, 93, 518.

[bibr9-10901981231177095] Blenner S. R. Roth S. E. Manukyan R. Escutia-Calderon Y. Chan-Golston A. M. Owusu E. Rice L. N. Prelip M. L. (2021). Community partnerships and experiential learning: Investing in the next generation of a diverse, qualified public health workforce. Pedagogy in Health Promotion, 7(1 Suppl.), 51S–62S. 10.1177/23733799211046974

[bibr10-10901981231177095] Bonilla-Silva E. (1997). Rethinking racism: Toward a structural interpretation. American Sociological Review, 62(3), 465–480. 10.2307/2657316

[bibr11-10901981231177095] Bonilla-Silva E. (2012). The invisible weight of whiteness: The racial grammar of everyday life in contemporary America. Ethnic and Racial Studies, 35(2), 173–194. 10.1080/01419870.2011.613997

[bibr12-10901981231177095] Bonini S. M. Matias C. E. (2021). The impact of Whiteness on the education of nurses. Journal of Professional Nursing, 37(3), 620–625. 10.1016/j.profnurs.2021.02.009 34016322

[bibr13-10901981231177095] Bowleg L. (2017). Towards a critical health equity research stance: Why epistemology and methodology matter more than qualitative methods. Health Education and Behavior, 44(5), 677–684. 10.1177/1090198117728760 28891342

[bibr14-10901981231177095] Bowleg L. (2021). “The master’s tools will never dismantle the master’s house”: Ten critical lessons for black and other health equity researchers of color. Health Education and Behavior, 48(3), 237–249. 10.1177/10901981211007402 34080476

[bibr15-10901981231177095] Braveman P. Parker Dominguez T. (2021). Abandon “Race.” Focus on Racism. Frontiers in Public Health, 9, 1–8. 10.3389/fpubh.2021.689462 PMC8452910 34557466

[bibr16-10901981231177095] Brayboy B. M. K. J . (2005). Toward a Tribal Critical Race Theory in education. Urban Review, 37(5), 425–446. 10.1007/s11256-005-0018-y

[bibr17-10901981231177095] Cacari Stone L. Avila M. Duran B . (2021). El Nacimiento del Pueblo Mestizo: Critical discourse on historical trauma, community resilience and healing. Health Education & Behavior, 48(3), 265–275. 10.1177/10901981211010099 34080474

[bibr18-10901981231177095] Camangian P. R. Cariaga S. M. (2021). Social and emotional learning is hegemonic miseducation: Students deserve humanization instead. Race Ethnicity and Education, 25(7), 901–921. 10.1080/13613324.2020.1798374

[bibr19-10901981231177095] Camangian P. R. Stovall D. O. (2022). Bang on the system: People’s praxis and pedagogy as humanizing violence. Urban Education. 10.1177/00420859221092967

[bibr20-10901981231177095] Chandanabhumma P. P. Duran B. M. Peterson J. C. Pearson C. R. Oetzel J. G. Dutta M. J. Wallerstein N. B. (2020). Space within the scientific discourse for the voice of the other? Expressions of community voice in the scientific discourse of community-based participatory research. Health Communication, 35(5), 616–627. 10.1080/10410236.2019.1581409 30786730

[bibr21-10901981231177095] Chandanabhumma P. P. Narasimhan S. (2020). Towards health equity and social justice: An applied framework of decolonization in health promotion. Health Promotion International, 35(4), 831–840. 10.1093/heapro/daz053 31236575

[bibr22-10901981231177095] Chávez V. Turalba R. A. N. Malik S. (2006). Teaching public health through a pedagogy of collegiality. American Journal of Public Health, 96(7), 1175–1180. 10.2105/AJPH.2005.062950 16735640 PMC3222303

[bibr23-10901981231177095] Chowkwanyun M. (2011). The strange disappearance of history from racial health disparities research. Du Bois Review, 8(1), 253–270. 10.1017/S1742058X11000142

[bibr24-10901981231177095] Cross R. I. (2018). Commentary: Can critical race theory enhance the field of public health? A student’s perspective. Ethnicity and Disease, 28, 267–270. 10.18865/ed.28.S1.267 30116097 PMC6092171

[bibr25-10901981231177095] Davis L. P. (2021). Still climbing the hill: Intersectional reflections on brown and beyond (pp. 21–23). American Educational Research Association.

[bibr26-10901981231177095] Delgado R. (1989). Storytelling for oppositionists and others: A plea for narrative. Michigan Law Review, 87(8), 2411–2441.

[bibr27-10901981231177095] Delgado Bernal D . (1998). Using Chicana feminist epistemology in educational research. Harvard Educational Review, 68(4), 555–582.

[bibr28-10901981231177095] Delgado Bernal D. Villalpando O . (2002). An apartheid of knowledge in academia: The struggle over the “legitimate” knowledge of faculty of color. Equity and Excellence in Education, 35(2), 169–180. 10.1080/713845282

[bibr29-10901981231177095] Delima D. G. (2019). Making a case for a funds of knowledge approach to teaching and learning for first-generation college students. College Teaching, 67(4), 205–209. 10.1080/87567555.2019.1630355

[bibr30-10901981231177095] Ford C. L. Airhihenbuwa C. O. (2010). The public health critical race methodology: Praxis for antiracism research. Social Science and Medicine, 71(8), 1390–1398. 10.1016/j.socscimed.2010.07.030 20822840

[bibr31-10901981231177095] Ford C. L. Airhihenbuwa C. O. (2018). Commentary: Just what is critical race theory and what’s it doing in a progressive field like public health? Ethnicity & Disease, 28(Supp1.), 223. 10.18865/ed.28.S1.223 30116090 PMC6092167

[bibr32-10901981231177095] Ford C. L. Griffith D. M. Bruce M. A. Gilbert K. L. (Eds.) (2019). Racism: Science & tools for the public health professional. American Public Health Association Press. 10.2105/9780875533049

[bibr33-10901981231177095] Ford C. L. Takahashi L. M. Chandanabhumma P. P. Ruiz M. E. Cunningham W. E. (2018). Anti-racism methods for big data research: Lessons learned from the HIV testing, linkage, & retention in care (HIV TLR) study. Ethnicity and Disease, 28, 261–266. 10.18865/ed.28.S1.261 30116096 PMC6092168

[bibr34-10901981231177095] Freire P. (2000). Pedagogy of the oppressed (Thirtieth anniversary edition). Continuum.

[bibr35-10901981231177095] French B. H. Lewis J. A. Mosley D. V. Adames H. Y. Chavez-Dueñas N. Y. Chen G. A. Neville H. A. (2020). Toward a psychological framework of radical healing in communities of color. Counseling Psychologist, 48(1), 14–46. 10.1177/0011000019843506

[bibr36-10901981231177095] The Futures Initiative. (2020). The futures initiative: The 10 essential public health services (Issue September, pp. 1–9).

[bibr37-10901981231177095] Galea S. Vaughan R. D. (2019). Public health, politics, and the creation of meaning: A public health of consequence, July 2019. American Journal of Public Health, 109(7), 966–968. 10.2105/AJPH.2019.305128 31166747 PMC6603446

[bibr38-10901981231177095] García J.-L. Gee G. C. Jones M. (2016). A Critical Race Theory analysis of public park features in Latino immigrant neighborhoods. Du Bois Review, 13(2), 397–411. 10.1017/S1742058X16000187

[bibr39-10901981231177095] Gee G. C. Hing A. Mohammed S. Tabor D. C. Williams D. R. (2019). Racism and the life course: Taking time seriously. American Journal of Public Health, 109(Suppl. 1), S43–S47. 10.2105/AJPH.2018.304766 PMC6356137 30699016

[bibr40-10901981231177095] Golden S. D. Earp J. A. L. (2012). Social ecological approaches to individuals and their contexts: Twenty years of health education & behavior health promotion interventions. Health Education and Behavior, 39(3), 364–372. 10.1177/1090198111418634 22267868

[bibr41-10901981231177095] Goodman M. S. Plepys C. M. Bather J. R. Kelliher R. M. Healton C. G. (2020). Racial/ethnic diversity in academic public health: 20-year update. Public Health Reports, 135(1), 74–81. 10.1177/0033354919887747 31747339 PMC7119260

[bibr42-10901981231177095] Griffith D. M. Semlow A. R. (2020). Art, anti-racism and health equity: “Don’t ask me why, ask me how!” Ethnicity and Disease, 30(3), 373–380. 10.18865/ED.30.3.373 32742139 PMC7360189

[bibr43-10901981231177095] Gwayi-Chore M. C. Del Villar E. L. Fraire L. C. Waters C. Andrasik M. P. Pfeiffer J. Slyker J. Mello S. P. Barnabas R. Moise E. Heffron R. (2021). “Being a person of color in this institution is exhausting”: Defining and optimizing the learning climate to support diversity, equity, and inclusion at the University of Washington School of Public Health. Frontiers in Public Health, 9(April), 1–12. 10.3389/fpubh.2021.642477 PMC8082071 33937172

[bibr44-10901981231177095] Hagopian A. West K. M. Ornelas I. J. Hart A. N. Hagedorn J. Spigner C. (2018). Adopting an anti-racism public health curriculum competency: The University of Washington experience. Public Health Reports, 133(4), 507–513. 10.1177/0033354918774791 29847749 PMC6055294

[bibr45-10901981231177095] Hardeman R. R. Burgess D. Murphy K. Satin D. J. Nielsen J. Potter T. M. Karbeah J. Zulu-Gillespie M. Apolinario-Wilcoxon A. Reif C. Cunningham B. A. (2018). Developing a medical school curriculum on racism: Multidisciplinary, multiracial conversations informed by Public Health Critical Race Praxis (PHCRP). Ethnicity & Disease, 28(Supp1.), 271. 10.18865/ed.28.S1.271 30116098 PMC6092164

[bibr46-10901981231177095] Hardeman R. R. Homan P. A. Chantarat T. Davis B. A. Brown T. H. (2022). Improving the measurement of structural racism to achieve antiracist health policy. Health Affairs, 41(2), 179–186. 10.1377/hlthaff.2021.01489 35130062 PMC9680533

[bibr47-10901981231177095] Harris C. I. (1993). Whiteness as property. Harvard Law Review, 106(8), 1707–1791.

[bibr48-10901981231177095] Harvey M. (2020). How do we explain the social, political, and economic determinants of health? A call for the inclusion of social theories of health inequality within U.S.-based public health pedagogy. Pedagogy in Health Promotion, 6, 246–252. 10.1177/2373379920937719

[bibr49-10901981231177095] Harvey M. McGladrey M. (2019). Explaining the origins and distribution of health and disease: An analysis of epidemiologic theory in core Master of Public Health coursework in the United States. Critical Public Health, 29(1), 5–17. 10.1080/09581596.2018.1535698

[bibr50-10901981231177095] Hernandez S. G. Genkova A. Castañeda Y. Alexander S. Hebert-Beirne J. (2017). Oral histories as critical qualitative inquiry in community health assessment. Health Education and Behavior, 44(5), 705–715. 10.1177/1090198117728546 28892652

[bibr51-10901981231177095] Hill Collins P . (1990). Black feminist thought: Knowledge, consciousness, and the politics of empowerment. Routledge.

[bibr52-10901981231177095] Hite A. Carter A. (2019). Examining assumptions in science-based policy: Critical health communication, stasis theory, and public health nutrition guidance. Rhetoric of Health & Medicine, 2(2), 147–175. 10.5744/rhm.2019.1009

[bibr53-10901981231177095] Hooks B. (1990). Marginality as a site of resistance. In Ferguson R. Gever M. Minh-ha T. T. West C. Tucker M. (Eds.), Out there: Marginalization and contemporary cultures (pp. 341–343). MIT Press.

[bibr54-10901981231177095] Huber L. P. (2009). Disrupting apartheid of knowledge: Testimonio as methodology in Latina/o critical race research in education. International Journal of Qualitative Studies in Education, 22(6), 639–654. 10.1080/09518390903333863

[bibr55-10901981231177095] Huynh J. (2022). “Family is the beginning but not the end”: Intergenerational LGBTQ chosen family, social support, and health in a Vietnamese American Community Organization. Journal of Homosexuality, 70(7), 1240–1262. 10.1080/00918369.2021.2018879 35007487

[bibr56-10901981231177095] Iftikar J. S. Museus S. D. (2018). On the utility of Asian critical (AsianCrit) theory in the field of education. International Journal of Qualitative Studies in Education, 31(10), 935–949. 10.1080/09518398.2018.1522008

[bibr57-10901981231177095] Johnson-Jennings M. Billiot S. Walters K. (2020). Returning to our roots: Tribal health and wellness through land-based healing. Genealogy, 4(3), 91. 10.3390/genealogy4030091

[bibr58-10901981231177095] Jones C. P. (2018). Toward the science and practice of anti-racism: Launching a national campaign against racism. Ethnicity & Disease, 28(Supp1.), 231. 10.18865/ed.28.S1.231 30116091 PMC6092166

[bibr59-10901981231177095] Jones C. P. Laveist T. A. Lillie-Blanton M. (1991). “Race” in the epidemiologic literature: An examination of the American Journal of Epidemiology, 1921-1990. American Journal of Epidemiology, 134(10), 1079–1084. 10.1093/oxfordjournals.aje.a116011 1746518

[bibr60-10901981231177095] Jones J. M. (1988). Racism in Black and White. In Eliminating racism (pp. 117–135), Springer. 10.1007/978-1-4899-0818-6_6

[bibr61-10901981231177095] Kagawa-Singer M. (2000). Improving the validity and generalizability of studies with underserved U.S. populations expanding the research paradigm. Annals of Epidemiology, 10(8 Suppl.), S92–S103. 10.1016/S1047-2797(00)00192-7 11189098

[bibr62-10901981231177095] Komro K. A. Lang D. L. Walker E. R. Harper P. D. (2018). Integrating structural determinants into MPH training of health promotion professionals. American Journal of Public Health, 108(4), 477–479. 10.2105/AJPH.2018.304309 29513578 PMC5844413

[bibr63-10901981231177095] Krieger N. (2014). Got theory? On the 21st c. CE rise of explicit use of epidemiologic theories of disease distribution: A review and ecosocial analysis. Current Epidemiology Reports, 1(1), 45–56. 10.1007/s40471-013-0001-1

[bibr64-10901981231177095] Krieger N. (2020). Measures of racism, sexism, heterosexism, and gender Binarism for health equity research: From structural injustice to embodied harm—An ecosocial analysis. Annual Review of Public Health, 41(1), 41–426. 10.1146/annurev-publhealth-040119-094017 31765272

[bibr65-10901981231177095] Krieger N. Boyd R. W. Maio F. De Maybank A. (2021). Medicine’s privileged gatekeepers: Producing harmful ignorance about racism and health. https://www.healthaffairs.org/do/10.1377/forefront.20210415.305480/full/

[bibr66-10901981231177095] Ladson-Billings G. (1995). But that’s just good teaching! The case for culturally relevant pedagogy. Theory into Practice, 34(3), 159–165.

[bibr67-10901981231177095] Ladson-Billings G. Tate W. F. (1995). Toward a critical race theory of education. Teachers College Record, 97(1), 47–68.

[bibr68-10901981231177095] Laster Pirtle W. N . (2020). Racial capitalism: A fundamental cause of novel coronavirus (COVID-19) pandemic inequities in the United States. Health Education & Behavior, 47, 504–508. 10.1177/1090198120922942 32338071 PMC7301291

[bibr69-10901981231177095] LaVeist T. A. (1994). Beyond dummy variables and sample selection: What health services researchers ought to know about race as a variable. Health Services Research, 29(1), 1–16. 8163376 PMC1069985

[bibr70-10901981231177095] Leonardo Z. Porter R. K. (2010). Pedagogy of fear: Toward a Fanonian theory of “safety” in race dialogue. Race Ethnicity and Education, 13(2), 139–157. 10.1080/13613324.2010.482898

[bibr71-10901981231177095] Liburd L. C. Hall J. E. Mpofu J. J. Williams S. M. Bouye K. Penman-Aguilar A. (2019). Addressing health equity in public health practice: Frameworks, promising strategies, and measurement considerations. Annual Review of Public Health, 41(1), 417–432. 10.1146/annurev-publhealth-040119-094119 31900101

[bibr72-10901981231177095] Lightfoot A. F. Efird C. R. Redding E. M. (2021). Developing an antiracist lens: Using photography to facilitate public health critical race praxis in a foundational MPH course. Pedagogy in Health Promotion, 7(4), 317–326. 10.1177/23733799211045712

[bibr73-10901981231177095] Loder C. M. Minadeo L. Jimenez L. Luna Z. Ross L. Rosenbloom N. Stalburg C. M. Harris L. H. (2020). Bridging the expertise of advocates and academics to identify reproductive justice learning outcomes. Teaching and Learning in Medicine, 32(1), 11–22. 10.1080/10401334.2019.1631168 31293184

[bibr74-10901981231177095] Lucy L. Demszky D. Bromley P. Jurafsky D. (2020). Content analysis of textbooks via natural language processing: Findings on gender, race, and ethnicity in Texas U.S. History Textbooks. AERA Open, 6(3), 233285842094031. 10.1177/2332858420940312

[bibr75-10901981231177095] Maglalang D. D. Rao S. (2021). “Theory’s cool, but theory with no practice ain’t shit. . .”: Critical theories and frameworks to dismantle racism in social work education and practice. Advances in Social Work, 21(2–3), 672–689. 10.18060/24145

[bibr76-10901981231177095] Mannor K. M. Malcoe L. H. (2022). Uses of theory in racial health disparities research: A scoping review and application of public health critical race praxis. Annals of Epidemiology, 66, 56–64. 10.1016/j.annepidem.2021.11.007 34793963

[bibr77-10901981231177095] McSorley A.-M. M. Manalo-Pedro E. Bacong A. M. (2021). Doctoral students as agents for change: Shaping our public health training environment. Pedagogy in Health Promotion, 7(4), 299–303. 10.1177/23733799211042642

[bibr78-10901981231177095] Misra S. Etkins O. S. Yang L. H. Williams D. R. (2022). Structural racism and inequities in incidence, course of illness, and treatment of psychotic disorders among Black Americans. American Journal of Public Health, 112(4), 624–632. 10.2105/AJPH.2021.306631 35319958 PMC8961835

[bibr79-10901981231177095] Mitchell L. K. Watson M. K. Silva A. Simpson J. L. (2022). An interprofessional antiracist curriculum is paramount to addressing racial health inequities. Journal of Law, Medicine & Ethics, 50, 109–116. 10.1017/jme.2022.15 35244003

[bibr80-10901981231177095] Nazareno J. Yoshioka E. Adia A. C. Restar A. Operario D. Choy C. C. (2021). From imperialism to inpatient care: Work differences of Filipino and White registered nurses in the United States and implications for COVID-19 through an intersectional lens. Gender, Work and Organization, 28(4), 1426–1446. 10.1111/gwao.12657 34230784 PMC8251240

[bibr81-10901981231177095] Neely A. N. Ivey A. S. Duarte C. Poe J. Irsheid S. (2020). Building the transdisciplinary resistance collective for research and policy: Implications for dismantling structural racism as a determinant of health inequity. Ethnicity and Disease, 30(3), 381–388. 10.18865/ED.30.3.381 32742140 PMC7360186

[bibr82-10901981231177095] Pasick R. J. Kagawa-Singer M. Stewart S. L. Pradhan A. Kidd S. C. (2012). The Minority training program in cancer control research: Impact and outcome over 12 years. Journal of Cancer Education, 27(3), 443–449. 10.1007/s13187-012-0375-7 22661253 PMC4482227

[bibr83-10901981231177095] Patton L. D. (2016). Disrupting postsecondary prose: Toward a critical race theory of higher education. Urban Education, 51(3), 315–342. 10.1177/0042085915602542

[bibr84-10901981231177095] Perez J. Leonard W. R. Bishop V. Neubauer L. C. (2021). Developing equity-focused education in academic public health: A multiple-step model. Pedagogy in Health Promotion, 7(4), 366–371. 10.1177/23733799211045986

[bibr85-10901981231177095] Petteway R. J. (2020). LATENT//missing: On missing values, narrative power, and data politics in discourse of COVID-19. Health Education and Behavior, 47(5), 671–676. 10.1177/1090198120950194 32806932

[bibr86-10901981231177095] Petteway R. J. (2021). Poetry as Praxis + “Illumination”: Toward an epistemically just health promotion for resistance, healing, and (re)imagination. Health Promotion Practice, 22(1 Suppl.), 20S–26S. 10.1177/1524839921999048 33942645

[bibr87-10901981231177095] Petteway R. J. Mujahid M. Allen A. Morello-Frosch R. (2019). Towards a people’s social epidemiology: Envisioning a more inclusive and equitable future for social epi research and practice in the 21st century. International Journal of Environmental Research and Public Health, 16, 20. 10.3390/ijerph16203983 PMC6843593 31635327

[bibr88-10901981231177095] Posselt J. R. (2018). Trust networks: A new perspective on pedigree and the ambiguities of admissions. Review of Higher Education, 41(4), 497–521. 10.1353/rhe.2018.0023

[bibr89-10901981231177095] Price R. Skopec M. Mackenzie S. Nijhoff C. Harrison R. Seabrook G. Harris M. (2022). A novel data solution to inform curriculum decolonisation: The case of the Imperial College London Masters of Public Health. Scientometrics, 124, 1021–1037. 10.1007/s11192-021-04231-3

[bibr90-10901981231177095] Public Health Accreditation Board. (2022). Standards and measures for initial accreditation, Version 2022 (Issue February).

[bibr91-10901981231177095] Ramirez-Valles J. Neubauer L. C. Zambrana R. E. (2022). Inequity within: A call for inclusion of Latina/o/x scholars in faculty and leadership ranks in schools and programs of public health. Public Health Reports, 138, 386–388. 10.1177/00333549221077072 35289648 PMC10031822

[bibr92-10901981231177095] Ratima M. Martin D. Castleden H. Delormier T. (2019). Indigenous voices and knowledge systems—Promoting planetary health, health equity, and sustainable development now and for future generations. Global Health Promotion, 26(3 Suppl.), 3–5. 10.1177/1757975919838487 30964406

[bibr93-10901981231177095] Rodney R. (2016). Decolonization in health professions education: Reflections on teaching through a transgressive pedagogy. Canadian Medical Education Journal, 7(3), 10–18. PMC5342882 28344704

[bibr94-10901981231177095] Sabado-Liwag M. D. Manalo-Pedro E. Taggueg R. Bacong A. M. Adia A. Demanarig D. Sumibcay J. R. Valderama-Wallace C. Oronce C. I. A. Bonus R. Ponce N. A. (2022). Addressing the interlocking impact of colonialism and racism on Filipinx/a/o American health inequities. Health Affairs, 41(2), 289–295. 10.1377/hlthaff.2021.01418 35130069

[bibr95-10901981231177095] Sandifer M. Gibson E. Brant-Rajahn S. (2022). Antiracist andragogy in school counselor education and training. Dialogues in Social Justice, 7(1), 1236.

[bibr96-10901981231177095] Schucan Bird K. Pitman L . (2020). How diverse is your reading list? Exploring issues of representation and decolonisation in the UK. Higher Education, 79(5), 903–920. 10.1007/s10734-019-00446-9

[bibr97-10901981231177095] Seiler J. Hajat A. Khosropour C. M. Guthrie B. L. Balkus J. E. (2022). A novel curriculum review process to initiate the incorporation of anti-racist principles into epidemiology course work. American Journal of Epidemiology, 191, 1527–1531. 35695754 10.1093/aje/kwac105

[bibr98-10901981231177095] Shaw-Ridley M. Ridley C. R. (2010). The health disparities industry: Is it an ethical conundrum? Health Promotion Practice, 11(4), 454–464. 10.1177/1524839910375612 20689052

[bibr99-10901981231177095] Shelton R. C. Adsul P. Oh A. (2021). Recommendations for addressing structural racism in implementation science: A call to the field. Ethnicity and Disease, 31, 357–364. 10.18865/ed.31.S1.357 34045837 PMC8143847

[bibr100-10901981231177095] Smith L. T. (2012). Decolonizing methodologies: Research and indigenous people. Zed Books. 10.1177/135910530100600310 22049381

[bibr101-10901981231177095] Solórzano D. G. (2022). My journey to this place called the RAC: Reflections on a movement in critical race thought and critical race hope in higher education. International Journal of Qualitative Studies in Education, 36(1), 87–98. 10.1080/09518398.2022.2042613

[bibr102-10901981231177095] Solórzano D. G. Bernal D. D. (2001). Examining transformational resistance through a Critical Race and LatCrit theory framework: Chicana and Chicano students in an urban context. Urban Education, 36(3), 308–342. 10.1177/0042085901363002

[bibr103-10901981231177095] Solórzano D. G. Yosso T. J. (2001). Critical Race and LatCrit theory and method: Counter-storytelling Chicana and Chicano graduate school experiences. International Journal of Qualitative Studies in Education, 14(4), 471–495. 10.1080/09518390110063365

[bibr104-10901981231177095] Soon N. A. Akee R. Kagawa M. Morey B. N. Ong E. Ong P. Ponce N. Tanjasiri S. P. (2020). Counting race and ethnicity for small populations during the COVID-19 pandemic. AAPI Nexus Journal: Asian Americans and Pacific Islanders Policy, Practice, 17(1), 1–7.

[bibr105-10901981231177095] Stern A. M. (2005). Sterilized in the name of public health. American Journal of Public Health, 95(7), 1128–1138. 10.2105/AJPH.2004.041608 15983269 PMC1449330

[bibr106-10901981231177095] Stewart Q. T. (2003). Swimming upstream: Theory and methodology in race research. In White logic, White methods: Racism and methodologies (pp. 111–127). https://tatetalks.web.unc.edu/wp-content/uploads/sites/7733/2015/10/stewart-2011-swiming-upstream-theory-and-methodology-in-race-research.pdf

[bibr107-10901981231177095] Sullivan S. (2014). The hearts and guts of white people: Ethics, ignorance, and the physiology of white racism. Journal of Religious Ethics, 42(4), 591–611. 10.1111/jore.12074

[bibr108-10901981231177095] Sun Y. (2014). Rethinking public health: Promoting public engagement through a new discursive environment. American Journal of Public Health, 104(1), 6–13. 10.2105/AJPH.2013.301638 PMC3910054 24228674

[bibr109-10901981231177095] Sweet P. L. (2020). Who knows? Reflexivity in feminist standpoint theory and Bourdieu. Gender and Society, 34(6), 922–950. 10.1177/0891243220966600

[bibr110-10901981231177095] Tsai J. Lindo E. Bridges K. (2021). Seeing the window, finding the spider: Applying critical race theory to medical education to make up where biomedical models and social determinants of health curricula fall short. Frontiers in Public Health, 9, 1–10. 10.3389/fpubh.2021.653643 PMC8313803 34327185

[bibr111-10901981231177095] Tuck E. (2009). Suspending damages. Harvard Educational Review, 79(3), 409–428.

[bibr112-10901981231177095] Utt J. (2018). A case for decentering whiteness in education. Explorations in Ethnic Studies, 41(1–2), 19–34. 10.1525/esr.2018.411205

[bibr113-10901981231177095] Volpp L. (2000). American Mestizo: Filipinos and antimiscegenation laws in California. UC Davis Law Review, 33(795), 795–835. 10.1017/CBO9781139043298.008

[bibr114-10901981231177095] Wahbi R. Beletsky L. (2022). Involuntary commitment as “Carceral- Health Service”: From healthcare-to-prison pipeline to a public health abolition Praxis. The Journal of Law, Medicine & Ethics, 50, 23–30. 10.1017/jme.2022.5 35244001

[bibr115-10901981231177095] Walensky R. P. (2021, April 8). Media statement from CDC Director Rochelle P. Walensky, MD, MPH, on Racism and Health. CDC Newsroom. https://www.cdc.gov/media/releases/2021/s0408-racism-health.html

[bibr116-10901981231177095] Walters K. L. Johnson-Jennings M. Stroud S. Rasmus S. Charles B. John S. Allen J. Kaholokula J. K. Look M. A. de Silva M. Lowe J. Baldwin J. A. Lawrence G. Brooks J. Noonan C. W. Belcourt A. Quintana E. Semmens E. O. Boulafentis J . (2020). Growing from our roots: Strategies for developing culturally grounded health promotion interventions in American Indian, Alaska Native, and Native Hawaiian Communities. Prevention Science, 21, 54–64. 10.1007/s11121-018-0952-z 30397737 PMC6502697

[bibr117-10901981231177095] Walters K. L. Mohammed S. A. Evans-Campbell T. Beltrán R. E. Chae D. H. Duran B. (2011). Bodies don’t just tell stories, they tell histories: Embodiment of Historical Trauma among American Indians and Alaska Natives. Du Bois Review, 8(1), 179–189. 10.1017/S1742058X1100018X 29805469 PMC5967849

[bibr118-10901981231177095] Wang P. Meng Y. Y. Lam V. Ponce N. (2019). Green space and serious psychological distress among adults and teens: A population-based study in California. Health and Place, 56, 184–190. 10.1016/j.healthplace.2019.02.002 30797185

[bibr119-10901981231177095] Williams D. R. Collins C. (1995). US socioeconomic and racial differences in health. Annual Review of Sociology, 21(1), 349–386. 10.1146/annurev.so.21.080195.002025

[bibr120-10901981231177095] Yancey A. Kagawa-Singer M. Ratliff P. Valdez A. Jimenez L. Banks P. Stewart S. Roe K. Pasick R. (2007). Progress in the pipeline: Replication of the minority training program in cancer control research. Journal of Cancer Education, 21(4), 230–236. 10.1080/08858190701347820 PMC3800744 17542715

[bibr121-10901981231177095] Yang J. S. (2010). Contextualizing immigrant access to health resources. Journal of Immigrant and Minority Health, 12(3), 340–353. 10.1007/s10903-008-9173-z 18704685

[bibr122-10901981231177095] Yosso T. J. (2005). Whose culture has capital? A Critical Race Theory discussion of community cultural wealth. Race Ethnicity and Education, 8(1), 69–91. 10.1080/1361332052000341006

[bibr123-10901981231177095] Zidani S. (2021). Whose pedagogy is it anyway? Decolonizing the syllabus through a critical embrace of difference. Media, Culture & Society, 43(5), 970–978. 10.1177/0163443720980922

[bibr124-10901981231177095] Zuberi T. (2000). Deracializing social statistics: Problems in the quantification of race. The Annals of the American Academy of Political and Social Science, 568(1), 172–185. 10.1177/000271620056800113

